# A Novel Natural Product, KL-21, Inhibits Proliferation and Induces Apoptosis in Chronic Lymphocytic Leukemia Cells

**DOI:** 10.4274/tjh.2013.0381

**Published:** 2015-05-08

**Authors:** Aysun Adan Gökbulut, Mustafa Yaşar, Yusuf Baran

**Affiliations:** 1 İzmir Institute of Technology Faculty of Science, Department of Molecular Biology and Genetics, İzmir, Turkey; 2 Naturin Natural Products, İzmir, Turkey; 3 Abdullah Gül University Faculty of Life and Natural Sciences, Kayseri, Turkey

**Keywords:** apoptosis, Cell cycle arrest, Chronic lymphocytic leukemia, KL-21

## Abstract

**Objective:**

The aims of this study were to examine the cytotoxic and apoptotic effects of KL-21, a novel plant product (produced by Naturin Natural Products, İzmir, Turkey), on 232B4 chronic lymphocytic leukemia (CLL) cells and to determine the cytotoxic effects on healthy BEAS-2B human bronchial epithelial cells.

**Materials and Methods:**

The cytotoxic effect of KL-21 was determined by MTT cell proliferation assay. Changes in caspase-3 enzyme activity were measured using the caspase-3 colorimetric assay. Changes in mitochondrial membrane potential were determined using the JC-1 dye-based method. Annexin V-FITC/PI double staining was performed to measure the apoptotic cell population. Effects of KL-21 on cell cycle profiles of CLL cells were investigated by flow cytometry.

**Results:**

We detected time- and concentration-dependent increases in the cytotoxic effect of KL-21 on 232B4 CLL cells. However, we also showed that, especially at higher concentrations, KL-21 was less cytotoxic towards BEAS-2B healthy cells than towards CLL cells. Annexin-V/PI double staining results showed that the apoptotic cell population increased in 232B4 cells. Increasing concentrations of KL-21 increased caspase-3 enzyme activity and induced loss of mitochondrial membrane potential. KL-21 administration resulted in small increases in the percentage of the cells in the G0/G1 phase while it decreased the S phase cell population up to 1 mg/mL. At the highest concentration, most of the cells accumulated in the G0/G1 phase.

**Conclusion:**

KL-21 has a growth-inhibitory effect on 232B4 CLL cells. KL-21 causes apoptosis and cell cycle arrest at G0/G1.

## Abstract (tr)

**Amaç:**

Bu çalışmanın amacı yeni bitkisel bir ürün olan KL-21’in [Naturin doğal ürünler şirketi (İzmir, Türkiye) tarafından üretilen] 232B4 kronik lenfostik lösemi (KLL) hücreleri üzerindeki sitotoksik ve apoptotik etkilerinin araştırılmasıdır. Ayrıca, KL-21’in BEAS-2B sağlıklı insan bronşial epitelyum hücreleri üzerindeki sitotoksik etkisine de bakılmıştır.

**Gereç ve Yöntemler:**

KL-21’in sitotoksik etkisine MTT hücre çoğalma testiyle bakılmıştır. Kaspaz-3 enzim aktivitesindeki ve mitokondri membran potansiyelindeki değişimlere sırasıyla kaspaz-3 kolorimetrik testi ve JC-1 boyasına dayalı bir yöntem kullanılarak bakılmıştır. Apoptotik hücre popülasyonunu belirlemek amacıyla Anneksin 5-FITC/PI ikili boyama yöntemi kullanılmıştır. KL-21’in KLL hücrelerinin hücre siklusu üzerindeki etkilerine akım sitometresi ile bakılmıştır.

**Bulgular:**

KL-21’in KLL hücrelerinin çoğalması üzerine etkisi zamana ve doza bağımlı olarak artmıştır. Bununlar beraber, KL-21’in özellikle yüksek konsantrasyonlarda KLL hücreleri ile karşılaştırıldığında BEAS-2B hücreleri üzerinde daha az sitotoksik etki gösterdiği saptanmıştır. Anneksin-5/PI ikili boyaması 232B4 hücrelerinde apoptotik hücre popülasyonunun arttığını göstermiştir. KL-21’in artan konsantrayonları kaspaz-3 enzim aktivitesini arttırmış ve mitokondri membran potansiyelindeki kayıpları indüklemiştir. KL-21 1 mg/ml konsantrasyonuna kadar G0/G1 fazındaki hücrelerin yüzdesinde küçük artışlara ve S fazındaki hücre popülasyonunda ise azalmalara neden olmaktadır. En yüksek konsantrasyonda ise hücrelerin büyük bir çoğunluğu G0/G1 fazında birikmiştir.

**Sonuç:**

Elde edilen sonuçlar, KL-21’in 232B4 KLL hücreleri üzerinde büyümeyi inhibe edici bir etkisi olduğunu göstermiştir. Ayrıca, K-21 apoptozu indüklemekte ve G0/G1 fazında hücre siklusunun tutulumuna neden olmaktadır.

## INTRODUCTION

Chronic lymphocytic leukemia (CLL) is a monoclonal disorder characterized by an increase in the number of functionally deficient mature CD5+ B lymphocytes in the blood, bone marrow, lymph nodes, and spleen [1,2]. CLL is most commonly observed in Western countries and affects mainly older individuals [3,4]. The main purposes of CLL treatment are to reduce cancer progression and induce apoptosis while providing quality of life for patients. Treatment approaches for CLL include chemotherapy, radiotherapy, immunotherapy, and bone marrow transplantation (BMT) with high-dose chemotherapy [4,5]. Among these treatment options, purine analogs such as fludarabine and cladribine, alkylating agents like chlorambucil, and alkylating agent/anthracycline combinations are frequently used [6]. Rituximab, a chimeric anti-CD20 monoclonal antibody, and alemtuzumab, a monoclonal antibody against the CD52 antigen, have received attention in the treatment of CLL due to their increased specificity as compared to chemotherapy [7,8]. BMT has been reported to decrease mortality when it is applied early in the course of the disease [9]. However, BMT, especially allogeneic BMT, is not considered as an appropriate regimen for CLL patients since most CLL patients are older than 55 years [10,11]. Although all the methods used in the treatment of CLL are effective, none of the standard therapies are capable of completely eliminating CLL. On the other hand, these methods have distinct side effects in the treatment of CLL [5]. Moreover, CLL remains an incurable disease with conventional therapies due to development of relapse or refraction [12]. For these reasons, it is necessary to improve the treatments for relapsed or refractory CLL or to develop new therapeutic agents that are less toxic and more effective for the complete elimination of CLL cells. Use of plant-derived substances such as resveratrol and quercetin has been indicated in several studies for CLL treatment, either alone or in combination with other agents [13,14]. KL-21 is a novel agent of plant origin produced by Naturin Natural Products (İzmir, Turkey) for the treatment of CLL. Ethical committee approved this study. In the present study, we aimed to investigate the possible cytotoxic and apoptotic effects of KL-21 on 232B4 CLL cells. 

## MATERIALS AND METHODS

### Cell Lines and Culture Conditions and Chemicals

Human 232B4 CLL cells were kindly provided by Prof. Dr. Ander Rosen from Linköping University, Sweden. Healthy human BEAS-2B epithelial cells were obtained from Assist. Prof. Dr. Ali Çağır from İzmir Institute of Technology and were used as a positive control. The cells were grown and maintained in RPMI-1640 medium containing 10% fetal bovine serum and 1% penicillin-streptomycin at 37 °C in 5% CO2. A 10 mg/mL stock solution of KL-21 was prepared in DMSO and stored at -20 °C. The final concentration of DMSO did not exceed more than 0.1% in culture. KL-21 is a novel plant extract containing extracts from 21 plant species in different amounts. KL-21 is a mixture of Achillea millefolium (8.47%), Juniperus communis (7.62%), Thymus vulgaris (6.78%), Peganum harmala (6.78%), Curcuma longa (6.78%), Silybum marianum (6.78%), Urtica dioica (6.77%), Equisetum arvense (5.93%), Lavandula stoechas (5.93%), Zingiber officinale (5.08%), Fumaria officinalis (4.24%), Taraxacum officinale (4.24%), Rosmarinus officinalis (4.23%), Nigella sativa (4.23%), Cichorium endivia (3.39%), Viscum album (2.54%), Solidago virgaurea (2.54%), Hypericum perforatum (2.54%), Acorus calamus (1.69%), Valeriana officinalis (1.69%), and Melissa officinalis (1.69%).

### Measurement of Cell Growth by MTT Assay

Antiproliferative effects of KL-21 were determined by the MTT cell proliferation assay as described. The IC50 value (the drug concentration that inhibits cell growth by 50%) of KL-21 was calculated according to cell proliferation plots [15].

### Annexin-V/PI Double Staining

We determined the translocation of phosphatidylserine from the inner membrane to the outer cell membrane in order to examine the apoptotic effects of KL-21 on CLL cells. Initially, 1x106 cells were treated with increasing concentrations of KL-21 (0.001 to 1 mg/mL) for 48 h. After incubation, the cells were washed twice with cold phosphate buffered saline (PBS) and then homogenized with 1 mL of 1X binding buffer, and then 100 µL of this solution was added into glass tubes. Next, 5 µL of FITC annexin V and 5 µL of propidium iodide (PI) were added to the cell solutions. These samples were vortexed gently and then incubated for 15 min at room temperature in the dark. Afterwards, 400 µL of 1X binding buffer was added to each tube, and samples were analyzed by flow cytometry (BD FACSCanto Flow Cytometer, Belgium) within 1 h.

### Analysis of the Changes in Mitochondrial Membrane Potential

We examined the loss of mitochondrial membrane potential (MMP) in response to KL-21 in 232B4 cells with the JC-1 Mitochondrial Membrane Potential Detection Kit (Cayman Chemicals, USA). Briefly, the cells (1x106 cells/2 mL), induced to undergo apoptosis, were collected by centrifugation at 180 x g for 10 min. Supernatants were removed and pellets were homogenized with 300 µL of medium, and then 30 µL of JC-1 dye was added to the cells and the cells were incubated at 37 °C in 5% CO2 for 30 min. They were centrifuged at 400 x g for 5 min, supernatants were removed, and 200 µL of assay buffer was added to the pellets and vortexed. This step was then repeated. Afterwards, all pellets were homogenized with 320 µL of assay buffer and 100 µL from each sample was added to a 96-well plate as triplicates. In healthy cells, the aggregate red form has absorption/emission maxima of 560/595 nm, whereas in apoptotic cells, the monomeric green form has absorption/emission maxima of 485/535 nm. The plate was read in these wavelengths with a fluorescence ELISA reader (Thermo Varioskan Spectrum, Finland). The ratio of fluorescent intensity of JC-1 monomers to fluorescent intensity of JC-1 aggregates was calculated for each concentration as well as the untreated control sample. Relative changes in cytoplasmic/mitochondrial JC-1 were then determined [16].

### Analysis of Caspase-3 Activity

Changes in caspase-3 activity of the cells were examined with a caspase-3 colorimetric assay kit (BioVision Research Products, USA). In short, the cells (1x106 cells/2 mL/well), induced to undergo apoptosis by KL-21, were collected by centrifugation at 180 x g for 10 min. The cells were lysed by adding 50 µL of chilled Cell Lysis Buffer and incubated on ice for 10 min before centrifugation at 10,000 x g for 1 min. Supernatants were transferred to new Eppendorf tubes, and the reaction mixture was prepared in 96-well plates by adding 50 µL of 2X reaction buffer (containing 10 mM DTT), 50 µL of sample, and 5 µL of DEVD-pNA substrate and was incubated for 2 h at 37 °C in a CO2 incubator. At the end of this period, the plate was read under 405 nm wavelengths with an ELISA reader. The absorbance values were normalized to protein concentrations determined by the Bradford assay. 

### Cell Cycle Analysis

This technique is based on the determination of amounts of dsDNA by using PI and flow cytometry. Briefly, 1x106 cells/2 mL were treated with increasing concentrations of KL-21 for 48 h. After the incubation period, cells were collected by centrifugation at 260 x g for 10 min. Supernatants were removed and pellets were homogenized with 1 mL of cold PBS, and then the samples were put on ice. Afterwards, while the cells were slightly vortexed, 4 mL of cold ethanol was added to these cells, and then the mixture was put on ice. Cells fixed by this method were incubated overnight at -20 °C for analysis. The next day, the cells were centrifuged at 260 x g for 10 min, and supernatants were completely removed from the pellets. Pellets were homogenized with 1 mL of cold PBS and were centrifuged again at 260 x g for 10 min. Afterwards, cell pellets were homogenized with 1 mL of PBS containing 0.1% Triton X-100; 100 µL of RNase A (200 µg/mL) was then added to these cells and the mixture was incubated at 37 °C for 30 min. After this incubation period, 100 µL of PI (1 mg/mL) was added to the cells. These cells were incubated at room temperature for 15 min and were then analyzed by flow cytometry.

### Statistical Analysis

Statistical significance was determined via GraphPad Prism 6.0 software using 1-way analysis of variance (ANOVA) for MTT analyses and 2-way ANOVA for annexin V, MMP, caspase-3 activity and cell cycle analyses. P<0.05 was considered to be significant. MTT, annexin V, MMP, caspase-3, and cell cycle analysis results were shown as the means of 3 independent experiments (N).

## RESULTS

### Cytotoxic Effects of KL-21 on 232B4 CLL and BEAS-2B Epithelial Cells

**Figure 1:**
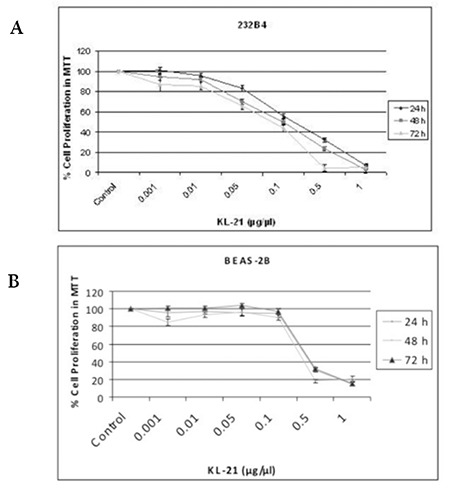
Cytotoxic effects of KL-21 on human 232B4 CLL cells (A) and BEAS-2B healthy cells (B) with statistical analysis. The IC50 value of KL-21 was calculated from cell proliferation plots. The results are the means of 3 independent experiments. The error bars represent the standard deviations. Statistical significance was determined using 1-way analysis of variance and p<0.05 was considered to be significant.

There were decreases in the viability/proliferation of 232B4 CLL cells in a dose- and time-dependent manner (Figure 1A), while KL-21 had very little or no effect on the viability of BEAS-2B healthy cells at up to 0.1 mg/mL (Figure 1B). Although KL-21 was cytotoxic toward healthy control cells at higher concentrations (0.5 and 1 mg/mL), more than 20% of cells were alive at indicated time points. IC50 values of KL-21 for 232B4 cells at 24, 48, and 72 h were calculated from cell proliferation plots and were found to be 0.2, 0.1, and 0.08 mg/mL, respectively. On the other hand, IC50 values of KL-21 for BEAS-2B cells were found to remain at about 0.4 mg/mL at each time point. These findings indicated that CLL cells are more susceptible than BEAS-2B cells to the growth inhibitory effects of KL-21.

### KL-21 Induced Apoptosis in a Dose-Dependent Manner in 232B4 Cells

**Figure 2:**
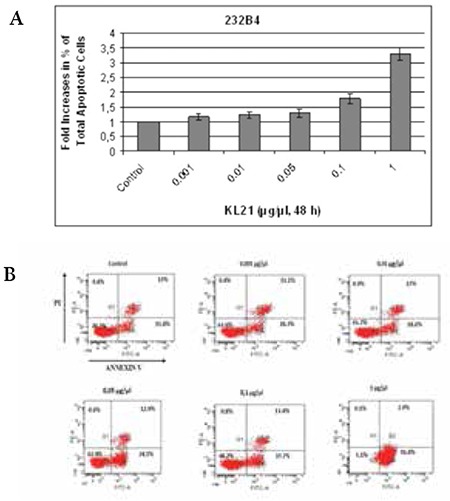
Evaluation of apoptosis in 232B4 cells induced by KL-21. The percentage of cells undergoing early and late apoptosis in a dose-dependent manner as compared to the control and flow cytometry analysis via annexin V-FITC/PI staining is shown (A and B). Cells in the lower right quadrant are annexin-positive/PI-negative early apoptotic cells. The cells in the upper right quadrant are annexin-positive/PI-positive late apoptotic cells. The percentage of cells annexin V-positive, PI-positive, or double positive for both annexin V and PI is indicated. The results are the means of 3 independent experiments.

The percentage of the apoptotic cell population (late apoptotic plus early apoptotic) of 232B4 cells was determined by flow cytometry. There were 1.16-, 1.23-, 1.28-, 1.77-, and 3.23-fold increases in percentage of apoptotic cells treated with 0.001, 0.01, 0.05, 0.1, and 1 mg/mL KL-21, respectively, when compared to untreated controls, as shown in Figures 2A and 2B. The data showed that KL-21 triggers apoptosis in a dose-dependent manner.

### KL-21 Induced Loss of Mitochondrial Membrane Potential in 232B4 Cells

**Figure 3:**
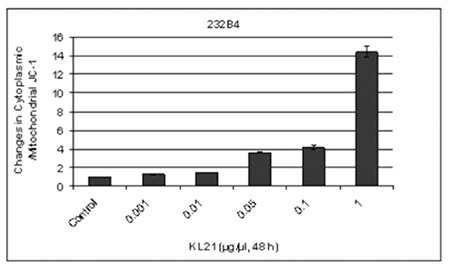
Percent changes in cytoplasmic/mitochondrial JC-1 in CLL cells treated with increasing concentrations of KL-21. The results are the means of 3 independent experiments. The error bars represent the standard deviations. Statistical significance was determined using 2-way analysis of variance and p<0.05 was considered to be significant.

There were 1.31-, 1.45-, 3.6-, 4.12-, and 14.4-fold increases in loss of MMP in 232B4 cells treated with 0.001, 0.01, 0.05, 0.1, and 1 mg/mL KL-21, respectively, as compared to untreated control cells (Figure 3).

### KL-21 Increased Caspase-3 Activity in 232B4 CLL Cells

**Figure 4:**
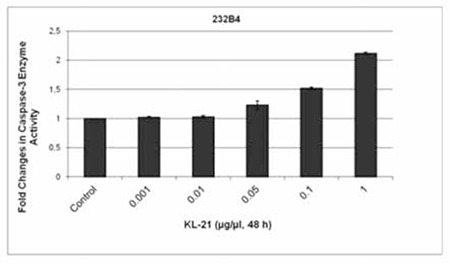
Changes in caspase-3 enzyme activity in response to KL-21. The results are the means of 3 independent experiments. The error bars represent the standard deviations. Statistical significance was determined using 2-way analysis of variance and p<0.05 was considered to be significant.

There were 1.02-, 1.03-, 1.23-, 1.52-, and 2.12-fold increases in caspase-3 enzyme activity in response to 0.001, 0.01, 0.05, 0.1, and 1 mg/mL KL-21, respectively, as compared to untreated controls (Figure 4).

### KL-21 Induced G0/G1 Arrest in 232B4 Cells

**Figure 5:**
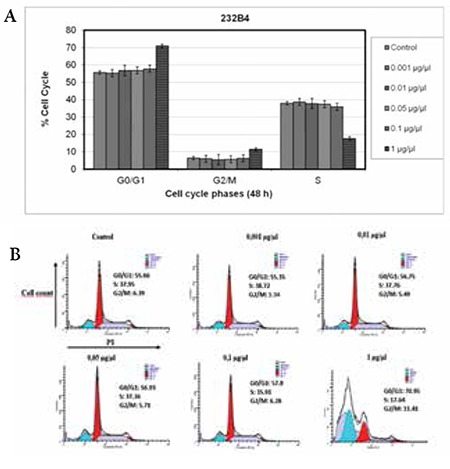
Effect of KL-21 on cell cycle progression. The percentage of cells in cell cycle phases are shown in the graph (A) and in quadrants (B). The results represent 3 independent experiments. Statistical significance was determined using 2-way analysis of variance and p<0.05 was considered to be significant.

In order to determine the possible mechanism of antiproliferative activity of KL-21, cell cycle progression of 232B4 cells was examined by flow cytometry in the presence of DNase-free RNase and PI dye. As summarized in Figures 5A and 5B, treatment of 232B4 cells with KL-21 resulted in small increases in the percentage of cells in the G0/G1 phase at 0.001 to 0.1 mg/mL, but there was a significant increase in response to 1 mg/mL KL-21.

## DISCUSSION

CLL is an adult leukemia characterized by accumulation of malignant B cells in several parts of the body. Despite the presence of many therapeutic regimens, CLL is still an incurable disorder [2]. CLL can be effectively treated with various agents; however, these strategies have their own side effects and some patients have limited therapeutic options [5,17]. Therefore, there is a need to discover novel drugs or agents for CLL treatment. 

We conducted this study to examine the cytotoxic and apoptotic effects of KL-21, a novel plant-derived product, on 232B4 CLL cells. KL-21 decreased proliferation of 232B4 CLL cells in a dose- and time-dependent manner. Moreover, we found that KL-21 did not have an antiproliferative effect on BEAS-2B cells at concentrations between 0.001 and 0.1 mg/mL. Although KL-21 was cytotoxic towards healthy cells, CLL cells were more susceptible to the cytotoxic effects of 0.5 and 1 mg/mL KL-21. On the other hand, annexin-V/PI double staining showed dose-dependent increases in the apoptotic cell population in response to KL-21 when compared to untreated controls. To elucidate the molecular mechanism of KL-21-induced apoptosis in CLL cells, we first checked the effect of KL-21 on MMP. The results revealed that KL-21 caused loss of MMP in a dose-dependent manner. In the literature, it is very well established that alterations in the structure and function of mitochondria play an important role in caspase-dependent apoptosis. Caspase-3, the executioner caspase, functions in the last step of caspase-mediated apoptosis. Therefore, we next checked the activation of caspase-3 enzyme in KL-21-treated 232B4 cells. Our data demonstrated that KL-21 increased caspase-3 activity in a dose-dependent manner when compared to untreated controls. Taken together, we can conclude that KL-21 induces apoptosis in 232B4 cells through the loss of MMP and caspase-3 activation. We also investigated the cytostatic property of KL-21 on CLL cells and observed that the treatment of CLL cells with KL-21 resulted in G0/G1 phase arrest of cell cycle progression, especially at high concentrations. Cells may either undergo repair or enter the apoptotic pathway following the G1 phase of the cell cycle to eliminate mutated or neoplastic cells. In the present study, we observed that CLL cells undergo significant apoptosis in response to KL-21 treatment. Therefore, it can be concluded that, in addition to the mitochondrial pathway of apoptosis, G1 phase arrest may be another mechanism of apoptosis in these cells, especially at high concentrations of KL-21.

By our group and some other groups it was reported that many plant-derived products have been shown to induce apoptosis in CLL cells. Honokiol, a plant product, triggers apoptosis in CLL cells via activation of caspase-3, -8, and -9, and apoptosis was further evaluated by annexin-V/PI double staining [18]. Gokbulut et al. showed that resveratrol and quercetin might block CLL growth by inducing apoptosis and cell cycle arrest [19]. The growth inhibition and induction of apoptosis in CLL cells treated with flavopiridol, which is isolated from the Indian plant Dysoxylum binectariferum, has been related to the downregulation of Bcl-2 [20].

KL-21 is a novel plant extract that is the herbal combination of 21 plant species at different percentages as described above. These plant species have been widely used in traditional medicine all over the world for the treatment of different diseases and some types of cancer. Most of them possess antiinflammatory, antiviral, antibacterial, antioxidant, and anticarcinogenic properties. Detailed information about the ingredients of KL-21 and their use in medicine is given in Table 1. As summarized in Table 1, terpenoids, flavonoids, and their derivatives are the main effective components of KL-21, similar to several plant-derived medicinal extracts. The main mechanisms of flavonoids include induction of apoptosis, cell cycle arrest, and inhibition of angiogenesis [49]. Terpenoids possess anticarcinogenic activities and their mechanisms of action include inhibition of NF-κB signaling, induction of apoptosis, and cell cycle arrest [50,51]. Other ingredients are generally plant-specific and their effects have been explained in several studies. For instance, the antitumor activity of hypericin from Hypericum perforatum has been shown to be related to the release of cytochrome c, activation of caspase-3, and partial inhibition of protein kinase C [52,53]. In conclusion, we demonstrated that KL-21 has growth-inhibitory effects on 232B4 CLL cells. KL-21 causes apoptosis and cell cycle arrest at G0/G1. While the complete mechanism of apoptosis needs to be elucidated, we have shown that KL-21 induced apoptosis by the mitochondrial/caspase-3-dependent pathway and the inhibition of cell cycle progression through the G0/G1 phase. Therefore, given its active plant constituents, the results of this study suggest that KL-21 could be a novel promising natural agent for the treatment of CLL. 

## Figures and Tables

**Table 1:**
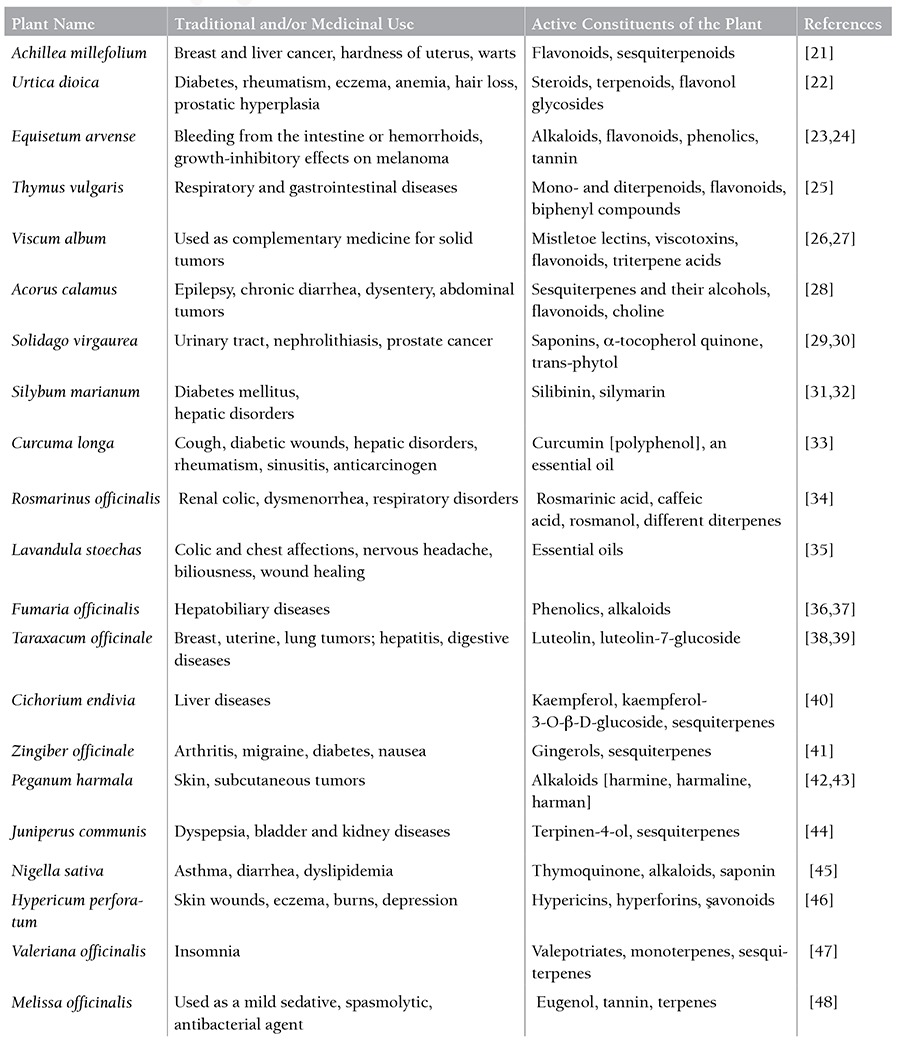
Botanical data of plant species included in KL-21.
